# Dead simple OWL design patterns

**DOI:** 10.1186/s13326-017-0126-0

**Published:** 2017-06-05

**Authors:** David Osumi-Sutherland, Melanie Courtot, James P. Balhoff, Christopher Mungall

**Affiliations:** 1 European Bioinformatics Institute (EMBL-EBI), Wellcome Trust Genome Campus, Cambridge, CB10 1SD UK; 2 RTI International, Research Triangle Park, 27709 NC USA; 3 Genomics Division, Lawrence Berkeley National Laboratory, Berkeley, 94720 CA USA

**Keywords:** OWL, OBO, Design pattern

## Abstract

**Background:**

Bio-ontologies typically require multiple axes of classification to support the needs of their users. Development of such ontologies can only be made scalable and sustainable by the use of inference to automate classification via consistent patterns of axiomatization. Many bio-ontologies originating in OBO or OWL follow this approach. These patterns need to be documented in a form that requires minimal expertise to understand and edit and that can be validated and applied using any of the various programmatic approaches to working with OWL ontologies.

**Results:**

Here we describe a system, Dead Simple OWL Design Patterns (DOS-DPs), which fulfills these requirements, illustrating the system with examples from the Gene Ontology.

**Conclusions:**

The rapid adoption of DOS-DPs by multiple ontology development projects illustrates both the ease-of use and the pressing need for the simple design pattern system we have developed.

## Background

Biologists classify biological entities in many different ways. A single neuron may be classified by structure (pseudo-bipolar), electrophysiology (spiking), neurotransmitter (glutamatergic), sensory modality (secondary olfactory neuron), location(s) within the brain (antennal lobe projection neuron, mushroom body extrinsic neuron), etc. A transport process occurring in a cell may be classified by the type of chemical transported, where transport starts and ends, and by what membranes are crossed. Bio-ontologies provide a widely used method for documenting such classifications and the relationships that apply between members of classes, such as partonomy. These classifications and relationships are central to the successful use of bio-ontologies in helping biologists make sense of the ever increasing volumes of data they work with. They are critical to the use of the Gene Ontology (GO) [1] and its associated annotations in interpreting genomic data via its application in enrichment analysis [2]. They are critical to the functioning of Virtual Fly Brain in grouping and querying neuroanatomical data [3].

To be successful in this role, bio-ontologies need to capture all of the many forms of classification that are important to biologists; but maintaining this manually becomes impractical as ontologies grow. Without formalization, the reasons for existing classifications are often opaque. The larger an ontology, the harder it is for human editors to find all valid classifications when adding a term, or to work out how to re-arrange the hierarchy when new intermediate classes are added.

The alternative to manually asserting classification is to use OWL inference to automate it. OWL equivalence axioms can be used to specify necessary and sufficient conditions for class membership. Standard reasoning software can then build a class hierarchy by finding classes that fulfill these conditions.

Many bio-ontologies now follow this approach, including the Uber Anatomy Ontology (Uberon) [4], the GO [5], the Ontology of Biomedical Investigations (OBI) [6], the Drosophila Anatomy Ontology (DAO) [7], the Cell Ontology (CL) [8] and the Ontology of Biological Attributes (Ontology of Biological Attributes (OBA) [9]. In the GO, over 52% of the classification is automated. Much of this classification leverages the structure of imported ontologies; for example, classification of transport processes in the GO relies on a classification of chemicals provided by the chemical ontology ChEBI [10] and on object property axioms specified in the OBO relations ontology.

A critical requirement for ongoing development of these ontologies is the specification of design patterns to guide the consistent OWL axiomatization required for automated classification. In many of these ontologies, classes are annotated with textual descriptions that follow standard patterns which also need to be documented. Where formal, machine-readable design patterns are sufficiently detailed, they can be used to quickly generate new classes, update old ones when a pattern changes, and automatically generate user-facing documentation.

### OWL design pattern systems

There is an extensive literature on ontology design patterns in OWL [11, 12]. Much of this is based on an approach known as Content Ontology Design Patterns (CODPs; see [12]) for an overview). CODPs are small, autonomous ontologies that specify multiple classes and properties. CODPs are typically re-used by one of two methods. Either the pattern is imported and new subclasses and sub-properties of pattern entities are instantiated in the target ontology, or it is used as a template, with entities in the pattern being given new identifiers in the namespace of the target ontology.

The GO and several other ontologies including CL and OBA already use standard patterns to generate new class terms via the TermGenie tool [13]. In GO, around 80% of new class terms are added via this route. This tool allows new terms to be added by specifying a desgin pattern and a set of fillers for variable slots. Unlike CODPs, these design patterns are not autonomous: they import classes and object properties from various ontologies. This means that their semantics are dependent on those of the ontologies they import from. This is by design: the patterns are intended to leverage classification and axiomatization from external ontologies to drive classification in the target ontology.

Design patterns in TermGenie are specified directly in Javascript. This specification is opaque to most human editors and is not easily reusable outside the context of TermGenie. The other major mechanisms for specifying design patterns for programmatic use are the languages Tawny OWL [14] and Ontology PreProcessing Language (OPPL) [15]. These are very powerful tools for generating and manipulating ontologies, but are not easy for ontology editors without strong technical backgrounds to write. They are also tied to specific languages and implementations, limiting their use.

Many editors of bio-ontologies are biologists with limited computational expertise beyond a basic understanding of some subset of OWL (typically limited to the subset of OWL that can be encoded in OBO 1.4 [16]), which they interact with via Manchester Syntax rendering and graphs in graphical editing tools such such as Protégé [17]. A simple, lightweight standard for specifying design patterns is needed in order to make their development and use accessible to these editors. This standard should be readable and editable by anyone with a basic knowledge of OWL. It must also be easy to use programmatically without the need for custom parsers – i.e. it should follow some existing data exchange standard that can be consumed by any modern programming language. Based on these requirements, we have defined a lightweight, YAML Ain’t Markup Language (YAML)-based syntax for specifying design patterns, called Dead Simple OWL Design Patterns, or DOS-DPs (inversion of two letters is an homage to the Web Ontology Language, OWL, on which it is based).

## Implementation

We have developed a formal specification of DOS-DPs using JSON-schema [18] draft 4 for use in validation and documentation. This is available from the DOS-DP repository [19], which also lists recommendations for additional validation steps. Description fields in the schema document intended usage. Where appropriate, the schema document also includes fields that document mappings to relevant OWL entities. We use the Python jsonschema package to validate the schema and test it against example patterns. Table 1 contains a summary of schema field types and how they are used.

**Table 1:** DOSDP JSON schema fields

Field type	Used to	Mandatory subfields	Optional subfields	Used in
Printf_owl	Specify a logical OWL axiom using printf to substitute variable values	axiom_type, text, vars	annotations	logical axioms
Printf_annotation	Specify an annotation using printf to substitute variable values	annotationProperty, text, vars	annotations	annotations
List annotation	Specify a list of annotation property axioms of a single type using a list of values specified by a data list variable	annotationProperty, value	-	annotations
Printf_owl_convenience	Specify a logical OWL axiom of a prespecified type, using printf to substitute variable values.	text, vars	annotations	equivalentTo, subClassOf, disjointWith, GCI
Printf annotation obo	Specify an annotation axiom of a prespecified type using a list of values specified by a data list variable	text, vars	annotations, xrefs	def, name, comment,
List_annotation_obo	Specify a list of annotation property axioms of a single type, pre-specified type. using a list of values specified by a data list variable	value	-	xrefs, exact_synonyms, …

*Field type*: Name of schema field type (JSON schema definition). *Used to*: Description of field usage. *Used in*: Schema Fields in which this field type is used

### Approach

DOS-DPs are designed to be easy to read, edit and parse. We chose YAML because it is relatively easy to read and write compared to other common data exchange formats such as JSON and XML, and can be consumed by a wide range of programming languages. In order to take advantage of JSON-Schema for specification and validation, DOS-DPs are restricted to the JSON compatible subset of YAML [20].

Each design pattern can have an arbitrary number of variables. For ease of reading, writing and parsing, variable interpolation uses printf, a standard part of most modern programming languages.

OWL is expressed using Manchester Syntax [21], the most human-readable and editable of the OWL syntaxes, and the one most editors with a basic knowledge of OWL are likely to have encountered. For ease of reading and editing, quoted, human-readable identifiers are used for OWL entities throughout the pattern. These are assumed to be sufficient to uniquely identify any OWL entity within a pattern. Dictionaries are used to map readable identifiers to compact URIs (CURIEs) – prefixed short form identifiers. A JSON-LD context is used to map these to full IRIs. The entity IRIs recorded in this way can be used to check reference ontologies to find the current validity and status of all entities referenced in a pattern.

While the full specification of DOS-DPs is intended to be generic and expressive, a major aim is to hide complexity from editors wherever possible. To this end, we define convenience fields that are suitable for use in common, simple design patterns. We also allow extensions that import and extend the core JSON schema and that specify default values for high level fields. For example, we define an extension to support the OBO standard. This defines convenience fields for expressing OBO standard annotations and specifies a default annotation property for readable identifiers and an OBO standard base URI pattern.

**Fig. 1:**
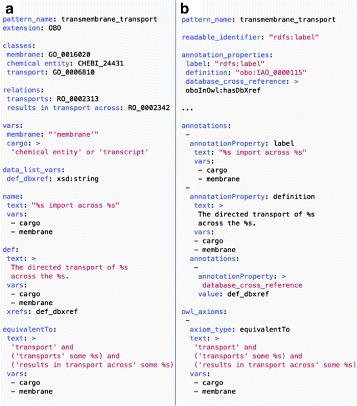
DOS-DP for defining classes of transmembrane import (based on an example from the GO.) *Panel A* shows the DOS-DP using the OBO extension. *Panel B* shows the same pattern expressed using the core specification (*classes*, *relations* and *vars* fields omitted from *panel B* for brevity). In *Panel A*, annotations are specified using dedicated fields (**def**, **name**, **xrefs**). The mapping from these to OWL annotation properties is specified in the OBO extension schema. This mapping is made explicit in *Panel B*, using an **annotation_property** dictionary and the **annotationProperty** field in axiom specifications under **annotations**. Throughout both versions of the pattern, paired fields **text** and **vars** specify printf text and fillers respectively. The value field is used with the **data_list_var** def_xrefs to specify a list database_cross_reference annotations on the definition

**Fig. 2:**
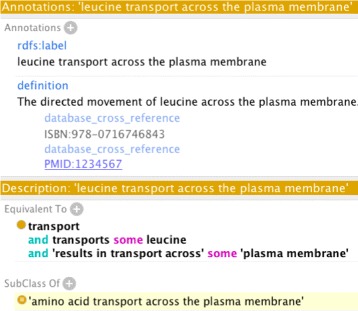
Example pattern implementation. An example of a term, ‘leucine transport across the plasma membrane’, generated using the pattern in Fig. 1. Note the automated classification under ‘amino acid transport across the plasma membrane’, specified using the same pattern

Figure 1 shows an example design pattern for generating classes of transport across a membrane defined by cargo type and membrane type. Figure 1 a shows a pattern following the OBO extension. Figure 1 b shows the same pattern expressed using the more verbose DOSDP core-specification. Figure 2 shows an example class generated using this pattern.

### Details

#### Pattern metadata

Each pattern is identified by an IRI. The short form of this IRI is recorded in a **pattern_name** field, and, by convention, is used for the file name. Each pattern optionally includes an extension specification, indicating the extension to be used in interpreting the pattern document. In 1 a this is set to OBO.

#### Dictionaries

In both versions of the pattern, the fields **classes** and **relations** serve as dictionaries for the OWL classes and object properties respectively used in the pattern, mapping human readable identifiers (keys) to short_form identifiers (values). The core pattern specifies an annotation property to use as a source of readable identifiers via the **readable_identifier** field. This is not required in the OBO extension version, as the extension specifies a default value of rdfs:label for this. The full pattern also contains an additional dictionary of OWL annotation properties. These are not required in the OBO extension, which specifies dedicated fields for annotation properties used in the OBO standard. The core DOSDP specification also defines a dictionary field for OWL data properties.

#### Input fields

All patterns contain one or more variable specification fields. These are simple objects in which the keys are variable names and the values specify variable range. The **vars** field specifies variables that range over OWL classes, specified as Manchester syntax expressions. For example, the value of the cargo variable in Fig. 1 is specified by the class expression: “‘chemical entity’ or ‘transcript”’. The quoted OWL entity names in this expression are specified in the dictionaries. Both patterns also include an example of a variable that takes a data type as an input. The **data_list_vars** field specifies variables whose values are lists in which all elements share an OWL data type, specified in the value of the variable field. For example def_dbxref in Fig. 1 is specified to be a list of (XSD) strings.

#### Output fields

The core schema has just two output fields: **annotations** for annotation property axioms and **logical_axioms** for logical owl axioms. The value of both of these fields is a list of axiom specifications. Each axiom specification includes a specification of axiom type (logical type or annotation property). Content is either specified using printf substitution of variable values into a text string (field type *printf_annotation* or *printf_owl* in Table 1 or by specifying a list of values to be used to generate multiple axioms of the same type (e.g. field type list_annotation in Table 1. Where OWL entities (specified as vars) are used to specify Printf substitution, the readable label of the entity is used. Axiom specifications can also be used to specify annotations of the specified axiom.

In our example, the annotations field is used to specify an rdfs:label axiom and a definition axiom. In both cases a text output is specified using a **text** field to specify a printf statement and a **vars** field to specify an ordered list of fillers. The definition axiom specification specifies a set of axiom annotations using a database_cross_reference annotation property. These axioms will be generated using a list of strings provided in the **data_list_var** **def_dbxref**. The results can be seen in Fig. 2.

The OBO version (1) encodes the same information using named fields: **name**, **def**, and **xrefs**. These fields follow the tag names used in OBO format [16]. The field specifications (in the OBO JSON schema doc) map these fields to the relevant OWL annotation properties, removing the need for ontology pattern developers to specify these mappings in an annotation property dictionary.

The **logical_axioms** field in Fig. 1 b specifies just one equivalence axiom. This is a very common pattern for defining classes. To make specifying this type of pattern easier, we define convenience fields that can be used whenever there is only one axiom of a given type per pattern. The pattern in 1 a uses the convenience field for **equivalentTo** to concisely capture the single logical axiom in this pattern.

## Discussion

### Limitations

DOS-DPs are designed to be simple and clear. There are a number of obvious ways that they could be made more powerful but which we have avoided in order to retain simplicity and clarity.

By design, DOS-DPs lack a mechanism for relating patterns to each other via inheritance or composition. Such mechanisms would add a technical burden to their, use requiring additional tooling, and so be a barrier to their adoption. Manual maintenance of design pattern hierarchies also risks re-creating the maintenance problem that these patterns are meant to solve.

For the sake of simplicity, DOS-DPs also lack a system for specifying optional clauses. This places some burden on the development of patterns that naturally form a subsumption hierarchy. However, the relationships between patterns can easily be derived by generating a set of OWL classes using default fillers (variable ranges) and classifying the results using a reasoner. This classification can then be used as a way of testing sets of DOS-DPs and to generate a browsable hierarchy of related patterns.

### Adoption

DOS-DPs are used both as formal documentation, and as part of the ontology-engineering pipelines in the GO, OBA, the Environmental Ontology (ENVO) [22], the Plant Trait Ontology [23], the Plant Stress and Disease Ontology [24], the Agriculture Ontology, and the Environmental Conditions and Exposures Ontology [25]; the central DOS-DP GitHub repo has a list of all adopters. See Figs. 1 and 2 for an example of a pattern used extensively in the GO.

One heavy user of (OPPL) patterns is Webulous, an application that allows specification of OWL classes using templates loaded into Google spreadsheets. It should be straightforward to develop a version of Webulous that supports design patterns specified as DOS-DPs, removing the need for expertise in OPPL to specify new patterns. Similarly, it should be possible to extend Tawny-OWL to support DOS-DPs. This could prove to be a very effective combination of accessible design pattern specification with a computationally powerful language for writing and manipulating OWL ontologies.

Patterns inevitably evolve as use-cases evolve. Changing all uses of an existing pattern by hand is impractical unless the number of uses is relatively low. For branches of ontologies where all terms follow a completely stereotyped pattern, we can specify whole branches simply by specifying a DOS-DP together with a URI and set of variable fillers for each term. We plan to use this to programmatically generate suitable branches of the GO at each release.

Where more flexibility is required, DOS-DPs could be used to update existing terms that are part of a human-edited ontology file. A system of tagging terms by the pattern they implement would allow all relevant terms to be identified. DOSDP-scala [26] can be used to identify existing classes within an ontology that follow a specified pattern, returning the fillers populating each variable in the pattern. If an ontology pattern changes then DOSDP-scala can also be used to test whether tagged terms conform to the old pattern, flagging those that do for automated update and those that do not for manual inspection.

## Conclusions

As can be seen from Fig. 1, which shows a pattern for defining terms in the GO, DOS-DPs are easy to read and write. The choice of YAML limits the need for balancing brackets and commas. The use of printf, Manchester syntax, and labels for OWL entities makes the pattern easy to read. Figure 2, which shows an application of the pattern specified in Fig. 1, illustrates how similar the pattern is to the way human editors interact with ontology classes in a GUI editor like Protégé [17]. As well as ease of reading and writing, our other aim is language independence. Currently there are partial (OBO-specific) implementations in Python [27] and Jython [28, 29], along with the Scala-based pattern matcher [26]. TermGenie is being extended to consume DOS-DPs. These implementations cover pattern validation and the addition of new classes. They also allow for generation of markdown format documentation from design patterns.

## Availability and requirements

**Project name:** Dead Simple OWL Design Patterns (DOS-DP). The specification and recommendations for validation are available from [29] under the GNU General Public License v3.0.

**Programming language and requirements:** The schema is specified using JSON-schema [18]. This specification can be consumed by any language for which a schema checker exists (see [18]).

## Abbreviations

ChEBI: Chemical entities of biological interest
CL: Cell ontology
CODP: content ontology design pattern
CURIE: Compact URI
DOS-DP: Dead simple OWL design pattern
GO: Gene ontology
GUI: Graphical user interface
IRI: Internationalized resource identifier
JSON: JavaScript object notation
OBA: Ontology of biological attributes
OBO: Open biomedical ontologies
OPPL: Ontology preprocessing language
OWL: Web ontology language
XML: Extensible markup language
XSD: XML schema description
YAML: YAML ain’t markup language
